# Second-generation antipsychotics—Cardiac ion channel modulation and QT interval disturbances: A review

**DOI:** 10.17305/bb.2025.13405

**Published:** 2025-12-11

**Authors:** Orhan Erkan, Ayse Suna Dai, Nihal Ozturk, Semir Ozdemir

**Affiliations:** 1 Department of Biophysics, Faculty of Medicine, Kafkas University, Kars, Türkiye; 2 Department of Biophysics, Faculty of Medicine, Akdeniz University, Antalya, Türkiye

**Keywords:** Second-generation antipsychotics, QT prolongation, torsades de pointes, hERG potassium channels, sodium channels, calcium channels, repolarization reserve, ventricular arrhythmias

## Abstract

Second-generation antipsychotics (SGAs) are frequently prescribed in psychiatry due to their efficacy and improved tolerability compared to first-generation agents. However, these medications are associated with significant cardiac adverse effects, particularly QT interval prolongation and torsades de pointes (TdP). This review aims to summarize the mechanisms by which SGAs affect cardiac ion channels and how these actions contribute to QT interval disturbances and increased arrhythmia risk. A narrative literature review was conducted using PubMed, Web of Science, and Google Scholar, without year restrictions, focusing on English-language experimental and clinical studies related to clozapine, olanzapine, risperidone, quetiapine, and ziprasidone. The findings indicate that all five SGAs inhibit the rapid delayed rectifier potassium current (I_Kr_) mediated by the human ether-a-go-go-related gene (hERG) potassium channel. Notably, the observed variability in the ratio of half-maximal inhibitory concentration to maximum free plasma concentration (IC_5__0_/C_max,free_) reflects its dependence on both the degree of hERG inhibition and the pharmacokinetic properties specific to each SGA. Additionally, several SGAs affect other potassium, sodium, and calcium currents, which may either mitigate or exacerbate the consequences of I_Kr_ inhibition. In conclusion, QT interval prolongation associated with SGAs is primarily driven by hERG potassium channel blockade, although the degree of this effect varies significantly among different agents. This variability highlights the necessity for electrocardiogram (ECG) monitoring and individualized cardiac risk assessments, especially for vulnerable patient populations.

## Introduction

Antipsychotic drugs represent an effective class of medications commonly prescribed for the treatment of psychiatric disorders, including schizophrenia and bipolar disorder [1, 2]. These medications are also utilized for various neuropsychiatric conditions, such as obsessive-compulsive disorder, delirium, and treatment-resistant depression [3, 4].

Antipsychotics are primarily categorized into two groups: typical or first-generation antipsychotics (FGAs) and atypical or second-generation antipsychotics (SGAs) [5]. FGAs, discovered in the 1950s, exhibit a high affinity for dopamine D2 receptors and significantly influence dopaminergic neurotransmission even at low doses [2, 6, 7]. The inhibition of dopamine release following FGA administration leads to a decrease in positive symptoms, such as delusions and hallucinations in the mesolimbic pathway, social withdrawal and loss of motivation in the mesocortical pathway, motor dysfunctions in the nigrostriatal pathway, and elevated prolactin levels in the tuberoinfundibular pathway [8–10]. FGAs are frequently associated with extrapyramidal side effects (EPS), including akathisia, dystonia, dyskinesia (tardive dyskinesia), and progressively developing parkinsonism [4, 11]. Additionally, they can cause endocrine-related adverse effects, such as galactorrhea, gynecomastia, and osteoporosis due to increased prolactin levels [3].

In contrast, SGAs are associated with a lower incidence of EPS and are generally better tolerated [3]. The discovery that clozapine has a low affinity for the D2 receptor facilitated the development of SGAs [12]. These drugs exhibit a broader mechanism of action by blocking not only dopamine D2 receptors but also serotonin 5-HT2A receptors [11]. Their enhanced interaction with serotonergic receptors and short-term binding and dissociation from the D2 receptor (often described as “kiss and run”) have contributed to their classification as atypical and have resulted in a reduced occurrence of EPS [11, 13]. Clinical studies have consistently demonstrated that SGAs enhance patient compliance and improve long-term treatment continuation rates compared to FGAs [14]. Their lower risk of EPS, combined with relatively greater therapeutic efficacy on negative symptoms, has made SGAs the preferred choice in clinical practice [2]. However, SGAs can also increase the risk of metabolic disorders, including weight gain, obesity, glucose intolerance, and dyslipidemia [15–17].

The side effects associated with antipsychotic drugs, which vary based on their receptor binding profiles, hold significant clinical relevance [18]. Antipsychotic medications display varying degrees of affinity for multiple receptors, and current research has yet to sufficiently clarify the specific receptors involved and the extent to which they alleviate psychosis. A comprehensive understanding of the pharmacodynamic and pharmacokinetic properties of these drugs is essential for accurately assessing their clinical efficacy and side effect profiles. Adverse effects from this class of medications may include restlessness, insomnia, sedation, dizziness, sexual dysfunction, obesity, metabolic syndrome, dyslipidemia, diabetes, and weight gain [3, 19].

While SGAs are noted for their improved tolerability and lower risk of EPS relative to FGAs, their potential cardiovascular effects must not be overlooked. These drugs are known to be associated with cardiac arrhythmias and an increased risk of sudden cardiac arrest. Such arrhythmias typically present with clinical manifestations, including QT interval prolongation (long QT syndrome; LQTS), ventricular tachycardia, and torsades de pointes (TdP) on electrocardiograms (ECGs). Corrected QT interval (QTc) prolongation induced by antipsychotic medications is particularly concerning, as epidemiological studies and case-control research have demonstrated an elevated risk of sudden death among psychiatric patients using these drugs [20–23]. Numerous studies have indicated that these medications can interact with ion channels regulating heart rhythm, resulting in various electrocardiographic changes [24–26]. Consequently, investigating the effects of SGAs on cardiac ion channels is critical for preventing potential cardiac complications and ensuring the safe administration of these drugs. This review aims to explore current findings regarding the modulation of ion channels as cellular mechanisms underlying the effects of SGAs on cardiac electrical activity. The analysis includes clozapine, olanzapine, risperidone, quetiapine, and ziprasidone, which are widely used second-generation antipsychotics associated with cardiac side effects in experimental and clinical studies. A comprehensive literature search was conducted in PubMed, Web of Science, and Google databases without year restrictions. All relevant publications with robust methodologies published in English were included. Keywords used during the literature search comprised “second-generation antipsychotics,” “QT prolongation,” “torsades de pointes,” “cardiac side effects,” “hERG channels,” “cardiac ion channels,” “sodium channels,” “potassium channels,” “L-type and T-type calcium channels,” “action potential,” “electrophysiology,” “patch-clamp,” and “cardiac myocytes.”

### Electrophysiological changes observed in second-generation antipsychotics

Cardiovascular diseases and psychiatric disorders are increasingly prevalent health concerns that significantly influence each other’s progression [27]. Autonomic nervous system disorders, often accompanying psychiatric conditions, along with genetic predispositions, smoking, unhealthy diets, and chronic inflammation, can adversely affect cardiovascular health. Conversely, stress and the physical and physiological consequences of lifestyle changes in individuals with cardiovascular disease may lead to the emergence of psychiatric symptoms or exacerbate existing disorders [28, 29]. Previous studies have demonstrated that individuals diagnosed with schizophrenia face a significantly higher risk of developing cardiovascular diseases compared to healthy individuals [30, 31].

While SGAs are generally considered safer than FGAs concerning EPS, they pose serious risks to cardiac electrophysiological markers. Consequently, their side effects have been extensively studied since their introduction. The use of SGAs has been shown to induce significant alterations in heart rhythm, autonomic nervous system function, and cardiac conduction. Common cardiac effects associated with SGAs include sinus tachycardia, QT interval prolongation, TdP, disturbances in heart rate variability (HRV), myocarditis, orthostatic hypotension, cardiomyopathy, and sudden cardiac death [32–36]. Notably, SGAs have been reported to cause more pronounced cardiac side effects than FGAs and may introduce new adverse effects unique to this class of drugs [37].

The QT interval, which spans from the beginning of the Q wave to the end of the T wave on an ECG, reflects the depolarization and repolarization of the ventricles. The QTc parameter, calculated using various formulas to account for individual variations in heart rates, provides a more definitive and reliable measurement [38]. Understanding the electrophysiological dynamics of the membrane that generates the cardiac action potential (AP) is crucial for evaluating physiological processes that influence the QT interval. Major membrane currents that can alter the QT interval include Na^+^ currents responsible for the AP depolarization phase, Ca^2+^ currents active during the plateau phase, and various K^+^ currents that govern the repolarization phase. Pathological changes in these currents or channels can lead to alterations in the QT interval on the ECG. Antipsychotic drugs primarily prolong the QT interval by blocking delayed-rectifier potassium currents (I_Kr_), also known as human ether-a-go-go-related gene (hERG) channels (K_v_11.1), which are responsible for the late repolarization of the AP [39, 40]. Such prolongation increases the risk of arrhythmias, including TdP—characterized as polymorphic ventricular arrhythmia—and ventricular fibrillation, which can ultimately result in cardiac arrest or sudden cardiac death [41, 42]. Table 1 summarizes the effects of SGAs (and selected metabolites) on ion channels, highlighting their inhibition of cardiac Na^+^ and Ca^2+^ currents alongside K^+^ currents (hERG/I_Kr_, I_to_, I_K1_) in heterologous systems and native myocytes.

**Table 1:** Inhibitory potency of second-generation antipsychotics on cardiac ionic currents/channels

**Drugs**	**Metabolite**	**Experimental** **model**	**IC_50_** **(µM)**	**Effect at [conc.]**	**Notes**	**Ref.**
Clozapine		CHO/hERG	2.8		Inhibition of hERG	[50]
		XO/hERG	28.3		Inhibition of hERG	[49]
		HEK-293	2.5		Inhibition of hERG	[49]
		Guinea-pig ventricular myocyte		24.7% [1 µM]	Inhibition of I_Kr_	[49]
		Guinea-pig ventricular myocyte		79.6% [5 µM]	Inhibition of I_Kr_	[49]
		Rabbit coronary arterial smooth muscle cells	7.84		Inhibition of I_Kv _	[52]
		HEK-293	23.7		Inhibition of I_CaT_	[54]
		CHO	9.2		Inhibition of I_CaL_	[51]
		HEK-293	10		Inhibition of I_Na_	[51]
		HEK-293	8.7		Inhibition of I_NaL_	[51]
		CHO	4.1		Inhibition of I_Kr_	[51]
		CHO	11		Inhibition of I_Ks_	[51]
		CHO	54		Inhibition of I_to_	[51]
		CHO	118		Inhibition of I_K1_	[51]
		Human atrial myocyte	51.7		Inhibition of I_to_	[25]
		Human atrial myocyte	28.2		Inhibition of I_Na_	[25]
		Human atrial myocyte	>100		Inhibition of I_K1_, I_sus_	[25]
		HEK-293	0.320		Inhibition of hERG	[25]
Olanzapine		HEK-293/hERG	8		Inhibition of hERG	[78]
		HEK-293/hERG	0.23		Inhibition of hERG	[81]
		CHO/hERG	6		Inhibition of hERG	[143]
		Human atrial myocyte	>100		Inhibition of I_to_, I_Na_, I_sus_ and I_K1_	[25]
		HEK-293	0.231		Inhibition of hERG	[25]
		CHO/hERG	27		Inhibition of hERG	[83]
	2-hydroxymethy l	HEK-293	11.6		Inhibition of hERG	[81]
	N-desmethyl	HEK-293	14.2		Inhibition of hERG	[81]
Risperidone		CHO/hERG	0.26		Inhibition of hERG	[99]
		Human atrial myocyte		29% [30 µM]	Inhibition of I_to_	[26]
		Human atrial myocyte		28.7% [3 µM]	Inhibition of I_sus_	[26]
		Human atrial myocyte		47.4% [30 µM]	Inhibition of I_sus_	[26]
		HEK-293	0.39		Inhibition of hERG	[144]
		HEK-293	25.5		Inhibition of I_to_	[144]
		CHO	9.7		Inhibition of I_Ks_	[144]
		CHO/hERG	1.6		Inhibition of hERG	[83]
		HEK-293		11% [8 nM]	Inhibition of hERG	[100]
		Rabbit ventricular myocyte		28% [1 µM]	Inhibition of I_Kr_	[106]
		Canine ventricular myocyte	0.92		Inhibition of I_Kr_	[107]
		HEK-293	0.148		Inhibition of hERG	[25]
		Human atrial myocyte	60		Inhibition of I_Na_	[25]
		CHO/hERG	0.167		Inhibition of hERG	[143]
		Guinea-pig ventricular myocyte	116		Inhibition of I_CaL_	[100]
Quetiapine		Rat ventricular myocyte		17% [10 µM]	Inhibition of I_to_	[127]
		Rat ventricular myocyte		18.6% [10 µM]	Inhibition of I_sus_	[127]
		Rat ventricular myocyte		12% [10 µM]	Inhibition of I_CaL_	[127]
		Rabbit coronary arterial smooth muscle cells	47.98		Inhibition of I_Kv_	[128]
		CHO/hERG	5.7		Inhibition of hERG	[143]
		HEK-293	8.3		Inhibition of hERG	[129]
		HEK-293	29.6		Inhibition of I_Na_	[130]
	Norquetiapine	HEK-293	10.8		Inhibition of hERG	[129]
	Norquetiapine	HEK-293	5.9		Inhibition of I_Na_	[130]
Ziprasidone		HEK-293	0.24		Inhibition of hERG	[141]
		CHO/hERG	0.169		Inhibition of hERG	[143]
		HEK-293	0.125		Inhibition of hERG	[25]
		Human atrial myocyte	>10		Inhibition of I_to_, I_K1_, I_Na_ and I_sus_	[25]

Abbreviations: CHO: Chinese hamster ovary; hERG: Human ether-a-go-go-related gene; XO: Xenopus oocytes; HEK-293: Human Embryonic Kidney-293.

Antipsychotic drugs vary significantly in their chemical structures and pharmacological properties. Due to their distinct receptor binding profiles, their effects on ion channels, QT interval, and AP differ. Thus, specific studies of each drug will enhance understanding of their cardiac side effects. In this context, we reviewed the effects of five commonly prescribed antipsychotic drugs on ion channels based on published research findings, discussing the potential cardiac risks associated with each.

### Pharmacological and electrophysiological characteristics of SGAs

SGAs share common therapeutic mechanisms but exhibit marked differences in their receptor binding affinities, metabolic characteristics, and effects on cardiac ion channels. These pharmacological and electrophysiological differences underpin both their clinical efficacy and distinct cardiac risk profiles, particularly regarding QT prolongation. Below, we summarize the key pharmacological and ion channel-related features of five widely used SGAs.

## Clozapine

**Figure 1.**
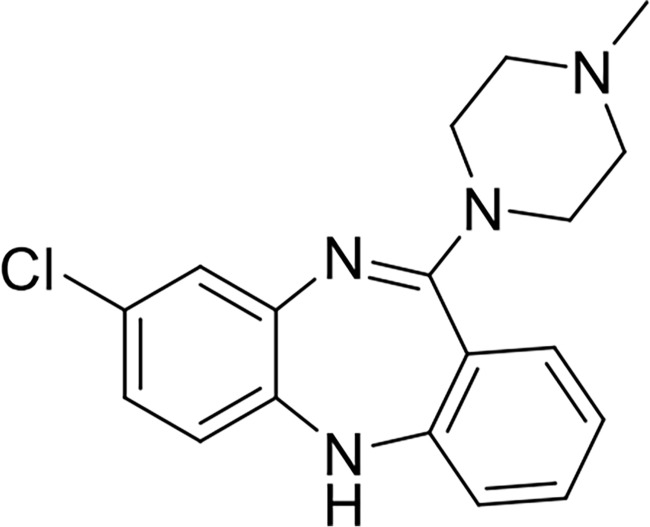
**Chemical structure of clozapine.** Clozapine is the first atypical (second-generation) antipsychotic, characterized by a tricyclic dibenzodiazepine scaffold structurally related to tricyclic antidepressants.

Clozapine, first discovered in 1959, is the inaugural atypical antipsychotic with a chemical structure resembling that of tricyclic antidepressants (Figure 1) [43]. A study conducted in Finland following its widespread use revealed a concerning incidence of agranulocytosis in 16 patients, with 8 fatalities due to severe infections [44, 45]. This report significantly curtailed clozapine’s use in Europe; however, it continued to be prescribed cautiously for patients resistant to other treatments. Its efficacy in ameliorating both positive and negative symptoms of psychosis, coupled with a lower risk of EPS compared to chlorpromazine, facilitated its continued use. After its reintroduction in 1989, the incidence of agranulocytosis decreased to 0.38% with careful monitoring in treatment-resistant schizophrenia [46]. Nonetheless, various side effects, including weight gain, diabetes, and myocarditis, have also been reported [16].

Clozapine’s complex receptor-binding profile contributes to both its clinical efficacy and side effect profile. Unlike typical antipsychotics, which primarily block D2 receptors, clozapine exerts its effects through serotonergic, noradrenergic, and glutamatergic receptors [5, 47]. This broad spectrum of pharmacological action is also reflected in its cardiac side effect profile. Notably, clozapine affects voltage-dependent ion channels in cardiomyocytes, leading to QT interval prolongation and increased susceptibility to potentially fatal ventricular arrhythmias. Real-world data suggest that QTc prolongation occurs more frequently in clozapine-treated patients than previously recognized. A 2024 Australian clinical study identified QTc prolongation in 36.5% of individuals receiving clozapine, significantly associating it with elevated heart rates and increased clozapine serum levels [48].

### Effects of clozapine on potassium currents

Clozapine is a potent antipsychotic medication used to treat specific patients with schizophrenia. However, its cardiac side effects, particularly those related to hERG channel inhibition and effects on K_v_ channels, merit careful consideration [49]. Clozapine has been shown to cause QT prolongation and increase the risk of TdP due to significant suppression of the I_Kr_ current through the inhibition of hERG channels [50].

The impact of clozapine on hERG channel activity has been extensively studied across various experimental models. Research involving Xenopus oocytes has demonstrated that clozapine inhibits hERG channels in a voltage-dependent manner, with half-maximal inhibitory concentration (IC_5__0_) values varying with applied voltage: 39.9 µM at --40 mV, decreasing to 28.3 µM at 0 mV, and further to 22.9 µM at +40 mV. Conversely, electrophysiological measurements on hERG channels expressed in HEK-293 cells revealed an IC_5__0_ value of 2.5 µM for clozapine [49]. Notably, the inhibition of hERG channels intensified at higher membrane potentials, indicating that clozapine binds more effectively to channels in their open or inactivated states [50]. This finding suggests that clozapine can inhibit hERG channels even at therapeutic plasma concentrations, significantly elevating the risk of QT prolongation [51]. In studies on guinea pig ventricular myocytes, a decrease of 24.7% in I_Kr_ current was recorded with 1 µM clozapine, escalating to 79.6% with 5 µM [49]. Furthermore, hERG currents were suppressed in both a voltage- and time-dependent manner in the presence of clozapine. The IC_5__0_ values obtained in this study indicate that even therapeutic doses can exert a considerable suppressive effect on hERG channels and I_Kr_, suggesting an increased risk of arrhythmias [49].

Understanding the binding and dissociation kinetics of medications is crucial for accurately assessing the risk of LQTS. A study investigating drug interaction kinetics with the K_v_11.1 potassium channel in Chinese hamster ovary (CHO) cells demonstrated that clozapine inhibits K_v_11.1 channels in a dose-dependent manner (IC_5__0_ ═ 2.8 µM). Additionally, a Markov model was developed to elucidate the binding and unbinding kinetics of clozapine [50]. This model included drug-bound open (O-D) and drug-bound inactivated (I-D) states alongside open (O) and inactivated (I) states. The dissociation constants (K_d_) for binding to the open and inactivated states were determined to be 1.46 µM and 1.63 µM, respectively. These findings accurately predicted the experimental IC_5__0_ values and clarified the kinetic structure of clozapine binding to K_v_11.1 channels [50]. The study highlights that the kinetic effects of conventional IC_5__0_ measurements may be overlooked, as drugs with slow binding and dissociation kinetics may exert greater effects at lower heart rates [50].

In a study involving rabbit coronary artery smooth muscle cells, clozapine was found to inhibit K_v_ channels in a concentration-dependent manner, with an IC_5__0_ value of 7.84 µM [52]. Analysis indicated that clozapine strongly inhibited the K_v_1.5 subtype while partially suppressing K_v_2.1 and K_v_7 channels [52]. Experiments designed to determine whether the inhibition was channel-specific demonstrated that clozapine exerted a specific effect on the K_v_1.5 channel subtype [53]. Following inhibition of K_v_2.1 and K_v_7 channels, the clozapine effect diminished from 45% to 21% and from 45% to 19%, respectively, indicating that these channels also significantly contribute to the effects of clozapine [52].

In summary, clozapine inhibits K_v_ channels in both cardiac myocytes and vascular smooth muscle cells, resulting in substantial alterations in cardiac and vascular physiology. These findings indicate that clozapine, as a SGA, necessitates careful monitoring, particularly in patients with cardiovascular conditions, as it exhibits significant ionic effects even at therapeutic doses [49–52]. However, it remains unclear whether clozapine affects transient outward potassium currents (I_to_), another K^+^ current that may influence QT duration.

### Effects of clozapine on calcium currents

The effects of clozapine on cardiac Ca^2+^ channels have also been thoroughly investigated, particularly its inhibitory actions on T-type Ca^2+^ channels (Ca_v_3.1, α1G) and L-type channels (Ca_v_1.2) [51, 54]. In a study by Choi and Rhim [54], clozapine was shown to inhibit Ca_v_3.1 currents in HEK-293 cells in a dose-dependent manner (IC_5__0_ ═ 23.7 µM). Additionally, the rate of inhibition of Ca_v_3.1 currents by clozapine varied in a voltage-dependent manner: at --100 mV, 10 µM clozapine inhibited Ca_v_3.1 current by 23% (IC_5__0_ ═ 23.7 µM), whereas at --75 mV, a more physiologically relevant voltage, the same concentration inhibited 52% (IC_5__0_ ═ 8.8 µM) [54]. This suggests that the inhibitory effect of clozapine on Ca^2+^ currents is stronger at more physiological voltage levels. Furthermore, channel inhibition has been reported to be use-dependent, with blockade occurring more rapidly at increased stimulation frequencies. Clozapine did not alter the activation kinetics of the channel but significantly slowed its deactivation, thereby enhancing Ca^2+^ influx [54]. Clozapine also markedly shifted the steady-state inactivation curve of Ca_v_3.1 channels to more negative potentials and delayed the inactivation kinetics. Given that Ca_v_3.1 channels are crucial in regulating cardiac pacemaker activity, this inhibitory effect has been linked to ventricular tachycardia, a notable cardiac side effect of clozapine [54]. Conversely, in a study by Le Marois et al. [51] involving CHO cells, clozapine was shown to inhibit Ca_v_1.2 L-type Ca^2+^ currents (I_CaL_), with an IC_5__0_ value of 9.2 µM. Therapeutic plasma concentrations of clozapine have been suggested to range from 1 to 4 µM [54–59], which is relatively low compared to the IC_5__0_ values. However, local concentrations may be more pronounced due to accumulation in cardiac tissue. Consistently, clozapine levels have been observed to be 10–24 times higher in the heart, liver, and brain tissues compared to plasma concentrations, indicating an effective concentration range of 10–100 µM under clinical conditions [54, 60]. Therefore, these findings suggest that clozapine can cause significant inhibition and kinetic modulation of Ca_v_3.1 channels even at clinically therapeutic concentrations, potentially resulting in cardiac side effects.

### Effects of clozapine on sodium currents

The impact of clozapine on cardiac ion channels has also been evaluated concerning voltage-gated sodium channel (Na_v_1.5) inhibition, demonstrating that it inhibits both peak and late current components of this channel [51]. In the study by Le Marois et al., dose-response curves were generated by applying clozapine at concentrations ranging from 0.12 µM to 100 µM, resulting in IC_5__0_ values of 8.7 µM for late sodium current (I_NaL_) and 10 µM for peak current [51]. Although clozapine is believed to reduce the depolarization rate of the AP due to the inhibition of Na^+^ channels, leading to slowed conduction in the myocardium, it is unlikely to cause significant blockade within the therapeutic dose range, given that therapeutic plasma concentrations are approximately 1–2 µM [51]. Additionally, clozapine inhibits not only the Na_v_1.5 channel but also repolarizing K^+^ currents, including K_v_11.1, whose blockade is a critical factor contributing to QT prolongation [50]. While clozapine may be expected to prolong AP duration (APD) and QT interval due to the blockade of repolarizing K^+^ currents, concurrent inhibition of depolarizing Na_v_1.5 and Ca_v_1.2 channels may counterbalance this effect [51].

A critical clinical factor to consider is that clozapine has been reported to accumulate in cardiac tissue [61]. This accumulation suggests that, while no significant inhibition of Na^+^ channels has been observed at therapeutic plasma concentrations, clozapine may cause cardiac conduction slowing at high doses or with prolonged use. This phenomenon may elucidate the association of clozapine with bradycardia and conduction defects [51]. Furthermore, clinical data indicate that although clozapine may induce QT prolongation, it does not significantly elevate the incidence of TdP [62]. It has been proposed that due to clozapine’s comparable inhibition of depolarizing and repolarizing currents, the prolongation of AP and QT interval will be minimal, resulting in a reduced risk of TdP. Consequently, despite clozapine’s inhibition of multiple cardiac ion currents, the balance among these currents suggests that its risk of TdP may be lower compared to other SGAs [51]. Nevertheless, given its potential for tissue accumulation, regular monitoring of patients receiving clozapine is still advisable.

## Olanzapine

**Figure 2.**
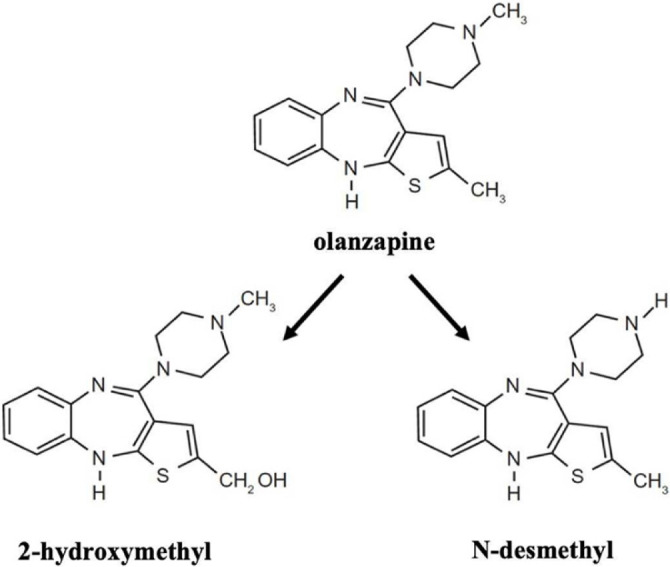
**Chemical structures of olanzapine and its metabolites.** Olanzapine and its major metabolites 2-hydroxymethyl-olanzapine and N-desmethyl-olanzapine are shown, all retaining the thienobenzodiazepine core that underlies the pharmacological similarity of olanzapine to clozapine.

Olanzapine is a clinically effective SGA that antagonizes dopamine and serotonin receptors [63]. Its chemical structure and major metabolites are illustrated in Figure 2. Pharmacologically and structurally, olanzapine is akin to clozapine. Unlike FGAs, olanzapine binds loosely to dopamine receptors, thereby facilitating normal dopamine neurotransmission. Approved for the treatment of schizophrenia in 1996, olanzapine is also widely employed for bipolar disorder and depression, with Food and Drug Administration (FDA) approval [11, 64]. Additionally, off-label applications include the treatment of acute agitation, delirium, anorexia nervosa, and chemotherapy-induced nausea and vomiting (CINV) [65–68]. *In vitro* studies demonstrate that olanzapine affects serotonergic (5-HT2A/C), dopaminergic (D1–4), histamine (H1), adrenergic (α1), and muscarinic (M1–5) receptors [69, 70].

Among the most prevalent adverse effects associated with olanzapine are increased appetite, significant weight gain, elevated blood glucose levels, and the onset of diabetes [15]. Additionally, similar to other atypical antipsychotics, olanzapine can lead to unpredictable cardiovascular effects [71]. These cardiovascular effects are generally associated with increased sympathetic activity, vagal inhibition, and QTc prolongation [72].

In one study, olanzapine use resulted in an average heart rate increase of 10 beats per minute as sympathetic activity predominated [73]. Olanzapine typically causes mild QTc prolongation; a clinical study found that the mean QTc prolongation in patients using olanzapine was approximately 10–15 ms [74]. In another study, QTc duration did not significantly prolong with olanzapine and generally remained below 500 ms [75]. The effects of olanzapine on cardiac repolarization have also been investigated in experimental animal models. A study conducted in anesthetized dogs revealed that olanzapine did not cause QT prolongation, though at high doses, it increased heart rate and induced vagal inhibition [76]. Recent inpatient data indicated that olanzapine was associated with QTc prolongation in 15.4% of patients [77]. Although the relative risk increase did not reach statistical significance, these findings suggest that olanzapine can significantly affect QTc in a subset of patients.

### Effects of olanzapine on potassium currents

A study examining olanzapine’s effects on hERG channels in HEK-293 cells demonstrated its concentration-dependent inhibition of hERG tail currents at --50 mV [78]. The IC_5__0_ for these currents was calculated to be 8.0 µM, a value significantly above therapeutic concentrations. Furthermore, olanzapine exhibited concentration-dependent inhibition (1 µM olanzapine 15.9%; 3 µM 31.7%; 10 µM 56.4%; 30 µM 78%; 100 µM 91.2%) along with increased channel activation, shifting the steady-state activation and inactivation curves toward more hyperpolarized potentials. hERG channel inhibition by olanzapine also displayed a voltage-dependent effect (--30 mV 31.4%; --10 mV 49.1%). However, at more depolarized potentials (between 0 and +50 mV), where channels are fully activated, olanzapine’s inhibition occurred independently of voltage. These findings indicate that hERG channel inhibition by olanzapine is voltage-, state-, and concentration-dependent [78]. Nonetheless, it has been reported that the IC_5__0_ value for olanzapine, with plasma concentrations ranging from 3.8 to 665.7 nM, is approximately 30 times higher than therapeutic levels [79]. However, olanzapine can accumulate in tissues up to 4–46 times the plasma level [78], with a heart/plasma ratio reaching 2.7 in guinea pigs [80], suggesting that hERG inhibition may become clinically relevant with long-term use or at elevated doses [78].

Another study in HEK-293 cells indicated that olanzapine and its metabolites (2-hydroxymethyl and N-desmethyl) blocked hERG current amplitude in a concentration-dependent manner, though to a lesser extent than thioridazine and sertindole [25]. The IC_5__0_ values for olanzapine, 2-hydroxymethyl, and N-desmethyl were calculated as 0.23 µM, 11.6 µM, and 14.2 µM, respectively [81]. A significant correlation was also observed between hERG blockade and QTc prolongation. At clinical plasma concentrations, olanzapine caused approximately 14% hERG blockade and led to a 1.7 ms QT prolongation, while 2-hydroxymethyl and N-desmethyl exhibited smaller effects on the QT interval [25, 82]. In another study conducted by Mow et al. [83] in CHO cells, olanzapine demonstrated dose-dependent I_Kr_ blockade on K_v_11.1, with an IC_5__0_ value of 27 µM, which was lower than other atypical antipsychotics (sertindole > haloperidol > risperidone > olanzapine). These results suggest that olanzapine is unlikely to trigger arrhythmias due to hERG inhibition, although it may have proarrhythmic potential at very high doses. Experiments conducted on human atrial cardiomyocytes to assess its effects on K^+^ channels showed that the IC_5__0_ value was >5 µM for I_to_, >10 µM for sustained potassium current (I_sus_), and inwardly rectifying potassium current (I_K1_). Based on these findings, it was concluded that olanzapine does not cause clinically significant inhibition, and therefore, the likelihood of cardiac risk from blockade of these channels is low [25]. Consequently, it is reasonable to assume that the observed QT prolongation associated with olanzapine is primarily related to hERG channel effects.

Lehmann and colleagues argued that estimating TdP risk based on QTc is not entirely reliable. Instead, the ratio of the hERG channel’s half-maximal inhibitory concentration (hERG IC_5__0_) to the peak serum concentration of unbound drug (C_max_) is commonly utilized in preclinical drug development to screen for compounds likely to induce TdP. In their meta-analysis employing the hERG IC_5__0_/C_max_ ratio, they found that this value for olanzapine was 1345, significantly above the threshold of 80, which indicates a negligible risk for TdP, suggesting that olanzapine poses no risk for TdP [84].

In summary, although olanzapine is an SGA that may not significantly affect the QT interval and has a low risk of inducing TdP, it should still be used cautiously at high doses or in patients with cardiac risk factors [25, 75, 76, 78, 83–86].

### Effects of olanzapine on sodium currents

Research indicates that olanzapine exerts minimal effects on Na^+^ channels. In myocytes derived from human right atrial tissues, olanzapine demonstrated a low capacity to block Na_v_1.5 channels, with an IC_50_ value exceeding 10 µM [25]. These results suggest that olanzapine does not significantly inhibit Na^+^ channels at clinically relevant concentrations, thereby presenting a low risk for Na^+^-current-related cardiac adverse effects. However, the limited number of studies on these channels necessitates further investigation to provide a definitive assessment of cardiac risks. Comprehensive studies employing diverse experimental models are essential. Additionally, the absence of experimental evidence regarding olanzapine’s effects on Ca^2+^ channels, which are crucial for the heart’s electrical and contractile activity, remains an area unaddressed in this review. Research addressing these gaps is critically important.

## Risperidone

**Figure 3.**
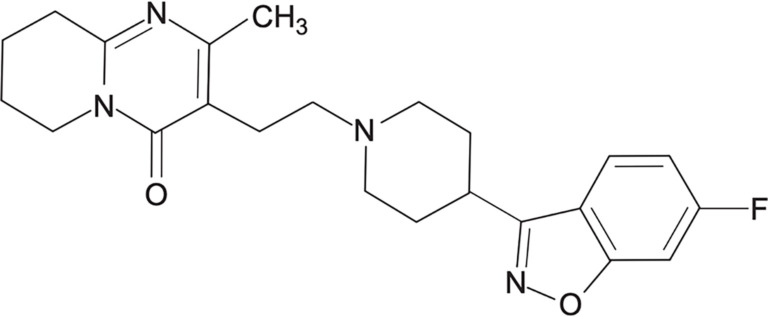
**Chemical structure of risperidone.** Risperidone is a benzisoxazole-derived second-generation antipsychotic that acts as a potent dopamine D2 and serotonin 5-HT2 receptor antagonist, and is widely used in the treatment of schizophrenia and acute bipolar disorder.

Risperidone, a benzisoxazole derivative and SGA, was approved by the FDA in 1993 (Figure 3). This medication strongly antagonizes dopamine D2 and serotonin 5-HT2 receptors [81, 87, 88]. It is primarily prescribed for the treatment of various psychotic disorders, including schizophrenia and acute bipolar disorder [89]. Moreover, risperidone has been associated with a significant reduction in the severity and incidence of EPS such as dystonia, akathisia, Parkinsonism, and tardive dyskinesia [90, 91].

Common dose-dependent adverse effects of risperidone include weight gain, hyperprolactinemia, sedation, dyslipidemia, hyperglycemia, and an increased risk of diabetes [15, 17]. Cardiovascular adverse effects may manifest as TdP resulting from QT prolongation, orthostatic hypotension, and tachycardia [92–94]. Extensive research on risperidone-related QT prolongation indicates that it prolongs QTc duration by an average of 20–30 ms in patients [95], with the risk of QT prolongation becoming particularly pronounced at higher doses. Reports have linked risperidone directly to QT prolongation and the subsequent risk of TdP [96], with significant QTc prolongation and TdP development observed in elderly patients [97]. Nonetheless, a 2025 cohort study found QTc prolongation in 8.45% of risperidone users when calculated using the Fridericia formula and 14.08% using the Bazett formula, although no cases exceeded 500 ms, and no serious arrhythmias were reported [98].

To better understand the cardiovascular changes and elucidate the electrophysiological mechanisms associated with QTc prolongation, a focused examination of the cardiac ionic currents involved in these physiological processes is warranted.

### Effects of risperidone on potassium currents

The impact of risperidone on cardiac electrophysiology has been extensively studied using various cellular and animal models. Its interaction with cardiac ion channels can induce significant alterations in the repolarization process. Notably, risperidone has been shown to cause QT prolongation through the inhibition of the K_v_11.1 channel [99] and to induce a marked delay in repolarization via strong inhibition of I_Kr_ [26, 100]. In experiments conducted on HEK-293 cells, the IC_5__0_ value for hERG channel inhibition by risperidone was determined to be 0.12 µM [99]. This finding indicates that risperidone can significantly inhibit hERG channels even at therapeutic plasma concentrations. In studies involving human atrial myocytes, a concentration of 5 µM of risperidone reduced I_Kr_ current by 60% and significantly prolonged APD [26].

In guinea pig papillary muscles, risperidone was found to prolong APD in a concentration-dependent manner. The administration of E-4031, a selective I_Kr_ blocker, along with chromanol 293B, a selective slow delayed rectifier potassium current (I_Ks_) blocker, led to a significant prolongation of AP duration. The greater impact of K^+^ channel blockers compared to risperidone alone suggests that this effect arises from the concurrent suppression of Ca^2+^ currents that counterbalance AP repolarization [24]. Additionally, in rabbit ventricular myocytes, risperidone prolonged APD in a concentration- and frequency-dependent manner; early afterdepolarization (EAD) waves were recorded in 6 out of 7 Purkinje fibers [101]. Besides its strong inhibitory effect on I_Kr_, risperidone has been shown to modulate G-protein-activated inwardly rectifying K^+^ (GIRK) channels [102]. While risperidone did not significantly affect I_K1_ current, it inhibited GIRK1/2 and GIRK1/4 channel currents expressed in Xenopus oocytes by 35% [102]. Meta-analyses evaluating the contribution of risperidone to TdP risk through hERG inhibition have calculated the hERG IC_5__0_/C_max_ ratio as 34 [84], suggesting that it should be classified as a high-risk drug for TdP. Clinical evidence supports that QT prolongation associated with risperidone use is pronounced, with documented clinical cases of TdP [103–105].

In an experimental study examining the electrophysiological effects of risperidone in CHO cells, it was demonstrated that, similar to other antipsychotic medications, risperidone inhibits I_Kr_ in a dose-dependent manner, with an IC_50_ value of 1.6 µM [83]. Conversely, in HEK-293 cells, Fossa et al. revealed that risperidone inhibited hERG current by 11% at a therapeutic concentration of 8 nM, with 20% inhibitory concentration (IC_20_) value of 11 nM, indicating relatively low values. The study aimed to show that the electrical alternans parameter (beat-to-beat variations in APD), measured in anesthetized guinea pig hearts, could be particularly useful in identifying medications with a higher risk of arrhythmias. The magnitude of alternans reflects the extent to which drugs affect the hERG channel and the potential for this effect to trigger arrhythmias. At a basic cycle length (BCL) of 200 ms, risperidone induced a minimal increase in alternans (less than 2 ms). However, at a BCL of 140 ms, alternans significantly decreased by 16 ms [100]. Consistent with these findings, although risperidone markedly inhibited hERG current in HEK-293 cells at high doses, no increase in alternans was observed; indeed, at high doses (74 times the clinical dose), alternans decreased. This suggests that risperidone may exhibit an antiarrhythmic tendency and that other protective mechanisms (e.g., autonomic nervous system modulation or intracellular Ca^2+^ dynamics) may contribute [100]. In ventricular cells isolated from New Zealand rabbits, the potential of risperidone to induce LQTS was assessed by measuring I_Kr_ inhibition, with 1 µM of risperidone reducing I_Kr_ tail current by 28%, identified as a primary mechanism responsible for APD prolongation. No effect on I_K1_ current was noted [106]. In a study by Magyar et al., a concentration-dependent decrease in I_Kr_ currents (IC_50_ ═ 0.92 µM) was observed in dog ventricular myocytes and guinea pig papillary muscles, while only a 9.6% reduction in I_Ks_ currents was noted at a high risperidone concentration of 10 µM [107]. They concluded that the observed prolongation of AP in both dog ventricular myocytes and guinea pig papillary muscles was related to reductions in these currents [107]. Furthermore, risperidone inhibited the hERG channel by 17% in HEK-293 cells, with an IC_50_ value of 0.148 µM [25]. The IC_50_ value of risperidone for I_to_ current was greater than 5 µM, and no clinically significant inhibition was detected. Similarly, the inhibition potentials for I_sus_ and I_K1_ were low, with IC_50_ values exceeding 10 µM. Consequently, the cardiac risk associated with the inhibition of I_to_, I_sus_, and I_K1_ channels by risperidone is suggested to be low [25].

In conclusion, risperidone does not cause a significant reduction in I_to_, I_sus_, or I_K1_ currents at clinical concentrations, indicating no substantial cardiac risk associated with the inhibition of these channels. However, it is important to emphasize that risperidone is a potent antipsychotic that significantly inhibits the hERG channel and suppresses the I_Kr_ current, thereby prolonging the QT interval and increasing the risk of TdP. Given the clear evidence of significant I_Kr_ current reduction at therapeutic doses, risperidone should be administered with caution, particularly in individuals with cardiovascular disease [24, 83, 84, 99–102, 104, 106].

### Effects of risperidone on calcium currents

The effects of risperidone on Ca^2+^ currents, alongside K^+^ currents, have been extensively studied [24, 100]. While it has been demonstrated to inhibit L-type Ca^2+^ channels in HEK-293 cells, its clinical significance at therapeutic plasma concentrations is minimal, primarily due to its high IC_50_ value of 116 µM [100]. A study on guinea pig papillary muscle indicated that risperidone caused a concentration-dependent prolongation of APD. Christ et al. examined the ionic currents contributing to this effect and observed a significant prolongation of AP with the application of I_Kr_ and I_Ks_ blockers (chromanol 239B and E-4031). Notably, the AP prolongation induced by risperidone was less pronounced compared to that caused by these blockers. Additionally, they demonstrated that risperidone decreases I_CaL_ in a concentration-dependent manner, suggesting a compensatory role in the prolongation of APD. These findings imply that risperidone’s effect on AP cannot be attributed solely to K^+^ channel inhibition but is also modulated by compensatory ionic mechanisms, such as L-type Ca^2+^ channel blockade. Consequently, the inhibition of I_CaL_ induced by risperidone emerges as the most plausible explanation for its limited effect on APD [24].

### Effects of risperidone on sodium currents

Although risperidone’s effects on cardiac electrophysiology are primarily associated with hERG channel inhibition, its impact on Na^+^ channels has also been investigated, revealing a low affinity for the Na_v_1.5 channel [25]. In this context, the IC_50_ value of risperidone on Na^+^ channels was found to exceed 10 µM in isolated human atrial cells [25]. These results suggest that clinically relevant Na^+^ channel inhibition by risperidone is unlikely. From a clinical standpoint, risperidone exhibits negligible effects on Na^+^ channels, and as such, it is not expected to significantly disrupt AP formation or conduction [25].

## Quetiapine

Quetiapine, a dibenzodiazepine-derived SGA, was developed in 1985 and received FDA approval in 1997 for the treatment of schizophrenia. It is also widely utilized for managing manic episodes in individuals with bipolar disorder and for bipolar depression, despite exhibiting relatively higher toxicity and mortality rates compared to other SGAs [108–111]. In numerous countries, quetiapine is prescribed off-label in combination with antidepressants for major depressive disorder [112]. Additionally, it is used off-label for various mental disorders, including anxiety disorder, delirium, psychotic disorders associated with dementia, and obsessive-compulsive disorder [113]. Its global preference is attributed to FDA approval and its versatility in treating schizophrenia, depression, and assorted psychiatric conditions.

**Figure 4.**
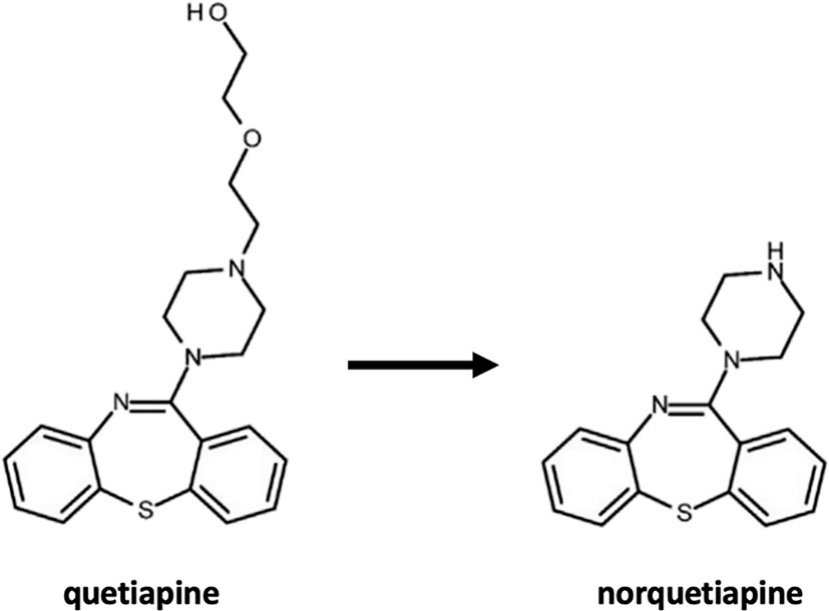
**Chemical structures of quetiapine and norquetiapine.** Quetiapine and its active metabolite norquetiapine are shown; despite their closely related structures, norquetiapine displays a distinct pharmacological profile, including inhibition of the norepinephrine transporter and partial agonist/antagonist actions at several serotonergic and adrenergic receptor subtypes.

Quetiapine is classified as a broad-spectrum SGA, demonstrating the ability to bind to multiple targets [114]. When compared to dopamine D1 and D2 receptors, quetiapine exhibits a notably higher binding affinity for 5-HT2A receptors within the serotonergic system [115, 116]. Furthermore, while it shows a strong propensity to bind to histamine and α-adrenergic receptors, its affinity for muscarinic receptors is comparatively lower [117, 118]. Quetiapine’s pharmacological effects vary across different dose levels, and its interactions with multiple receptors contribute to its side effect profile. Norquetiapine, the active metabolite of quetiapine, shares a similar chemical structure but elicits distinct pharmacological effects (Figure 4) [119, 120]. Notably, norquetiapine inhibits the norepinephrine transporter (NET) and exhibits partial antagonist effects on 5-HT2C, 5-HT7, α2, and 5-HT1A receptors [121, 122].

Common adverse effects associated with quetiapine include persistent somnolence, orthostatic hypotension, and dizziness [123, 124]. It has been linked to weight gain and disturbances in lipid metabolism, as well as increased cardiovascular risk factors, such as sinus tachycardia, myocarditis, cardiomyopathy, and QTc prolongation [125]. Real-world data from 2024 indicate that quetiapine is associated with a modest mean QTc increase of approximately 18 ms, with severe QT prolongation (QTc > 500 ms or ΔQTc > 60 ms) occurring in 13% of users. These patients exhibited significantly higher rates of ventricular arrhythmias and sudden cardiac death, underscoring that quetiapine-related QT risk is clinically significant in vulnerable populations [126].

### Effects of quetiapine on potassium currents

Although quetiapine is regarded as a less potent hERG inhibitor compared to other SGAs, some studies have demonstrated its significant impact on cardiac repolarization [127]. Research by Erkan et al. revealed that 10 µM quetiapine reduced the I_to_ current density by 17%, while a concentration of 100 µM resulted in a 23% reduction. Moreover, the I_sus_ current density was significantly diminished at both low and high concentrations [127]. While the potential for quetiapine to prolong the QT interval has been largely attributed to hERG channel inhibition, these findings suggest that its effects on potassium currents, such as I_to_ and I_sus_, may also contribute to QT prolongation [127]. However, further experimental and clinical studies are required to validate quetiapine’s effects on human cardiac physiology.

In smooth muscle cells derived from the coronary arteries of male New Zealand rabbits, quetiapine was shown to inhibit K_v_ channels in a concentration-dependent manner (48% inhibition at +60 mV, IC_50_ ═ 47.98 µM). Although quetiapine did not alter the steady-state activation curve, it induced a negative shift in the steady-state inactivation curve. To identify which subtypes of channels contribute to the total K_v_ current and which of these subtypes may mediate the effects of quetiapine, selective inhibitors of K_v_1.5 (DPO-1), K_v_2.1 (guangxitoxin), and K_v_7 (linopirdine) were employed. Following pretreatment with DPO-1 and linopirdine, quetiapine exhibited similar inhibition, whereas the presence of guangxitoxin markedly diminished this additional effect, indicating that K_v_1.5 and K_v_7 are not the primary targets (44% and 43% inhibition in the presence of DPO-1 and linopirdine, respectively), while K_v_2.1 appears to play a partial role (34% inhibition in the presence of guangxitoxin) [128].

In another investigation focusing on hERG potassium currents, both quetiapine and norquetiapine were found to inhibit these currents in a concentration-dependent manner (IC_50_ ═ 8.3 and 10.8 µM, respectively). At high potentials where the channel is fully open, norquetiapine’s effect was voltage-independent, and the steady-state inactivation curve of hERG currents shifted to more hyperpolarized potentials in its presence. Consequently, it was postulated that quetiapine and norquetiapine exhibit comparable potency in inhibiting hERG tail currents [129].

### Effects of quetiapine on calcium currents

Research on the effects of quetiapine on Ca^2+^ channels within the cardiovascular system is scarce, with only one study available [127]. This investigation, conducted on Wistar rat ventricular myocytes, revealed that quetiapine significantly inhibited ICaL by 12% at a concentration of 10 µM and by 21% at 100 µM [127]. Notably, while low concentrations did not significantly alter inactivation kinetics, higher concentrations dramatically prolonged the inactivation time (τ value increased from 95 ms to 189 ms). Consistent with these results, quetiapine was found to directly impact the contractile activity of ventricular myocytes [127]. The slowed inactivation of the channel, particularly at elevated concentrations, may contribute to this effect. These findings support the hypothesis that quetiapine directly affects L-type Ca^2+^ channels, thereby reducing the contractility of ventricular myocytes. However, further experimental and clinical studies are necessary to elucidate the underlying mechanisms and confirm quetiapine’s effects on the human heart.

### Effects of quetiapine on sodium currents

When examining quetiapine’s effects on cardiac Na^+^ channels, its active metabolite, norquetiapine, was shown to effectively inhibit Na_v_1.5 channels [130]. In this study, Kim et al. utilized HEK-293 cells expressing the Na_v_1.5 channel and thoroughly evaluated the effects of both quetiapine and norquetiapine on Na^+^ channels under various experimental conditions. The effect of quetiapine on Na_v_1.5 was found to be voltage-dependent, with an IC_50_ value of 504.8 µM at a holding potential of --120 mV and 29.6 µM at --90 mV [130]. These findings suggest that quetiapine is unlikely to induce clinically significant Na^+^ channel blockade. Conversely, norquetiapine exhibited much lower IC_50_ values, measured at 36.8 µM at --120 mV and 5.9 µM at --90 mV. Furthermore, norquetiapine has been shown to block the Na_v_1.5 channel with higher affinity, particularly in the inactivated state [130]. This suggests a mechanism akin to that of antiarrhythmic drugs. Its use-dependent blockade (increasing with stimulation frequency) implies that it may lead to conduction defects, especially in cardiac cells stimulated at higher frequencies. Active metabolites of antipsychotics may exhibit similar or different pharmacodynamic and pharmacokinetic properties compared to their parent compounds; they may be responsible for all or part of the therapeutic effects and may even reverse or neutralize specific activities. This is primarily due to norquetiapine’s stronger binding to the inactivated state of Na_v_1.5 compared to quetiapine, which slows recovery from inactivation. Norquetiapine shifts the channel’s steady-state inactivation to a more negative potential, facilitating the transition to the inactivated state and enhancing the blockade’s effectiveness. Furthermore, norquetiapine’s lower 1/K*_i_* value (affinity for binding to the inactivated state) indicates that the drug may remain in the channel longer (dissociating slowly), thereby reinforcing the blocking effect [130].

## Ziprasidone

**Figure 5.**
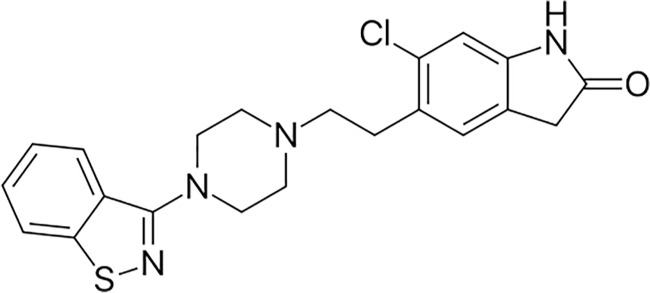
**Chemical structure of ziprasidone.** Ziprasidone is a benzisothiazolylpiperazine-derived second-generation antipsychotic with markedly higher affinity for serotonin 5-HT2A than dopamine D2 receptors, a profile that supports its atypical antipsychotic properties and relatively lower risk of extrapyramidal side effects.

Ziprasidone is a second-generation antipsychotic approved by the FDA in 2001 and is structurally classified as a benzisothiazolylpiperazine derivative (Figure 5). Like other atypical antipsychotics, ziprasidone strongly antagonizes dopamine D2 and serotonin 5-HT2 receptors. However, it exhibits an eightfold higher affinity for the 5-HT2A receptor compared to the D2 receptor [131]. This selectivity enhances the antipsychotic effect while mitigating the risk of EPS. Additionally, its 5-HT1A agonist activity alleviates negative symptoms by increasing dopamine levels in the frontal cortex and reduces EPS associated with D2 antagonism [132]. Ziprasidone induces a sedative effect through moderate affinity binding to histamine-1 receptors and can cause orthostatic hypotension by interacting with alpha-1-adrenergic receptors [131].

Ziprasidone has been approved by the FDA for treating acute manic or mixed episodes linked to schizophrenia and bipolar disorder [133]. It is considered safe as it does not produce clinically significant metabolic side effects and has minimal to no impact on prolactin levels or anticholinergic side effects [134]. However, literature regarding its cardiovascular side effects presents conflicting results. A study by Timour et al. suggested that ziprasidone carries a high risk of QTc prolongation and TdP development. It has been reported to prolong the QTc interval by an average of 20–30 ms in patients, with a dose-dependent effect [95]. Recent clinical data confirm that ziprasidone poses the highest risk of QTc prolongation among commonly used second-generation antipsychotics, aligning with preclinical findings that indicate strong hERG channel blockade as a basis for its proarrhythmic potential [135, 136]. Conversely, some studies indicate that ziprasidone does not induce TdP even at high doses exceeding 160 mg/day, with no arrhythmias observed in cases of intentional overdose [137, 138]. Nonetheless, the use of antipsychotics, including ziprasidone, has been associated with increased mortality in dementia-related psychosis, prompting the FDA to issue a “Black Box” warning for this population. A warning was subsequently added to the product label, advising against its use in patients with serious cardiovascular disease, electrolyte imbalances, or those taking medications that may prolong the QT interval [139]. To address these uncertainties, a logical and scientific approach involves examining ionic currents, which can yield a more comprehensive understanding of the mechanisms underlying QT interval alterations.

### Effects of ziprasidone on potassium currents

Ziprasidone, similar to other antipsychotics, has been postulated to prolong the QT interval by inhibiting the hERG current. Despite its consistent inhibitory potency on hERG in experimental settings, clinical data regarding cardiac adverse events remain inconclusive [47, 140]. Electrophysiological studies conducted on HEK-293 cells have demonstrated that ziprasidone inhibits the hERG channel in a concentration-dependent manner, with inhibition levels of 22.1%, 41.4%, 64.7%, and 80.8% at concentrations of 0.01, 0.1, 1, and 10 µM, respectively (IC_50_ ═ 0.24 µM). These results indicate that ziprasidone is a potent hERG channel inhibitor [141].

Furthermore, another investigation reported that ziprasidone ranks among the most potent hERG inhibitors, following sertindole [25]. The IC_50_ value for the hERG channel was observed to be quite low at 0.125 µM, causing a 22.7% blockade at plasma concentrations and leading to a 15.9 ms prolongation of the QT interval. Lehmann et al. [84] also reported an IC_50_ value of 169 nM and a C_max_ of 1.64 nM for hERG channels expressed in HEK-293 cells. They suggested that ziprasidone poses a minimal risk for TdP, as the hERG IC_5__0_/C_max_ ratio, which serves to assess TdP induction risk, was determined to be 103 [84]. In this context, ziprasidone was found to carry a lower TdP risk than antipsychotics such as risperidone (4.13) and haloperidol (51), yet a higher risk than olanzapine (1345). Conversely, in human atrial myocytes, I_to_, I_sus_, and I_K1_ currents were evaluated, revealing an IC_50_ value for I_to_ exceeding 5 µM, a level not associated with clinically significant inhibition [25]. Likewise, the IC_50_ values for I_sus_ and I_K1_ currents were found to exceed 10 µM, reinforcing the notion that ziprasidone is unlikely to cause clinically significant inhibition through these channels. Taken together, these findings suggest that the cardiac risks associated with ziprasidone are primarily attributable to hERG channel blockade.

### Effects of ziprasidone on sodium currents

The effects of ziprasidone on I_NaL_ are particularly noteworthy. Wu et al. [142] demonstrated that ziprasidone may heighten proarrhythmic potential by augmenting I_NaL_. In their study, monophasic APD (MAPD_90_) was measured in cells harvested from the left ventricular wall of female New Zealand rabbits, assessing the effects of ziprasidone alongside ATX-II toxin to increase I_NaL_. Ziprasidone significantly prolonged MAPD_90_ at a concentration of 1 µM, with more pronounced effects observed when administered with ATX-II [142]. These results underscore the need for caution in ziprasidone use, particularly in patients with low repolarization reserve, as increased I_NaL_ may lead to intracellular Na^+^ and Ca^2+^ overload, potentially triggering arrhythmias such as EAD and ventricular tachycardia [142].

Another study investigating ziprasidone found it to have an inhibitory effect on Na_v_1.5 in cells isolated from human atrial myocytes, although the IC_50_ exceeded 10 µM [25]. These findings suggest that clinical doses of ziprasidone are unlikely to significantly inhibit the Na^+^ channel. Specifically, ziprasidone demonstrates minimal capacity to block the fast Na^+^ current that initiates APs, indicating it is not expected to substantially affect the fast depolarization phase. Clinically, no evidence has been found to suggest that ziprasidone induces cardiac conduction delays due to Na^+^ channel blockade [25].

## Conclusion

SGAs exhibit distinct electrophysiological effects on ion channels within cardiac myocytes. This class of medications primarily prolongs the cardiac repolarization phase through the inhibition of potassium currents (I_Kr_, specifically the hERG channel current). A degree of hERG channel blockade has been documented across all SGAs, leading to prolonged APD and QT interval, which may initiate potentially fatal ventricular arrhythmias such as TdP. However, there are significant variations in the affinity for the hERG channel and the extent of blockade among different SGAs. For instance, clozapine, risperidone, and ziprasidone demonstrate potent I_Kr_ inhibition at low concentrations, significantly suppressing the I_Kr_ current even at therapeutic doses, potentially resulting in QT prolongation. Conversely, SGAs like olanzapine and quetiapine exhibit low hERG channel inhibition, thereby presenting a reduced risk of proarrhythmia associated with I_Kr_ blockade. In general, while SGAs may decelerate repolarization via the hERG channel, the risk of QT prolongation and arrhythmias varies among agents at clinical doses.

The data presented in Table 2 illustrate that the affinities of SGAs for the hERG channel significantly differ from the free drug concentrations achieved at therapeutic doses. Such variations elucidate the disparity in clinical QT prolongation risk among agents, as the relevant metric is not merely the IC_5__0_ value, but rather its relationship to the circulating free drug concentration. Consequently, the IC_5__0_/C_max,free_ ratio serves as a vital pharmacological safety index, estimating the likelihood of hERG channel inhibition under therapeutic conditions. An IC_5__0_/C_max,free_ ratio exceeding 30 indicates a substantial safety margin for hERG channel inhibition, whereas a low ratio signifies a considerable alteration in repolarization and a heightened risk of QT prolongation. This framework facilitates a more integrated interpretation of the electrophysiological repercussions of SGAs on cardiac ion channels.

**Table 2:** Comparison of hERG inhibition potency and pharmacokinetic parameters of SGAs

**Drug**	**IC_5__0_ (hERG, µM)**	****∼**f_u_**	****∼**C_max_, _total_ (µM)**	****∼**C_max, free_ (µM)**	****∼**IC_5__0_/C_max, free_**
**Clozapine**	0.32–28.3	0.05	1.53	0.076	4.2–370
**Olanzapine**	0.23–27	0.07	0.50	0.035	6.6–771
**Risperidone**	0.14–1.6	0.15	0.02	0.003	42–481
**Quetiapine**	5.7–10.8	0.17	2.09	0.36	15.8–30
**Ziprasidone**	0.12–0.24	0.01	0.79	0.008	15–30

Abbreviations: IC_50_: Half-maximum inhibitory concentration of the hERG channel; f_u_: Unbound fraction (1-protein binding); C_max_,_total_: Peak serum concentration; C_max, free_: Peak unbound concentration; SGAs: Second-generation antipsychotics; hERG: Human ether-a-go-go-related gene potassium channel.

The effects of SGAs on cardiac ion currents beyond hERG were generally observed at higher concentrations and are of lesser clinical significance at therapeutic levels. Most SGAs do not markedly inhibit I_to_, I_K1_, and I_sus_. For instance, both risperidone and ziprasidone block I_to_ and I_K1_ currents at high concentrations, with IC_5__0_ values exceeding 5–10 µM, suggesting that the functionality of these channels is likely unaffected at therapeutic doses. Clozapine has been shown to inhibit certain K_v_ channels, a subset of potassium channels, at micromolar levels, although the clinical relevance of this effect remains uncertain. Regarding calcium channels, studies indicate that SGAs can influence L-type (Ca_v_1.2) and T-type (Ca_v_3.1) Ca^2+^ currents. Risperidone is reported to block L-type Ca^2+^ channels solely at high doses (IC_50_ ≈ 10 µM), with negligible effects at therapeutic plasma levels. In contrast, clozapine is believed to inhibit Ca_v_3.1 only at elevated concentrations; however, studies on HEK-293 cells have shown significant inhibition of the Ca_v_3.1 channel within the 1–10 µM range, an effect that intensifies at physiological membrane potentials [54]. It has been posited that inhibition of T-type Ca^2+^ currents, which are involved in cardiac pacemaker activity, may correlate with ventricular tachycardia, a recognized cardiac side effect of clozapine. Additionally, certain SGAs exert a blocking effect on the fast Na^+^ current (Na_v_1.5), which initiates the early phase of APs. Nevertheless, this effect is typically limited at therapeutic concentrations and is not anticipated to result in clinically significant conduction delays. Quetiapine and its active metabolite, norquetiapine, block Na^+^ channels only at high concentrations during repetitive stimulation, suggesting they do not exert a significant effect on the human heart under therapeutic conditions [130]. Although clozapine can inhibit both the peak and late components of Na^+^ channels, this effect is not likely to induce conduction defects during routine treatment, given its IC_50_ value of approximately 8–10 µM. Due to the limited number of electrophysiological studies, definitive conclusions regarding ziprasidone’s effects on cardiac ion channels are not yet possible. The relative scarcity of research on its impact on sodium and potassium currents, coupled with the lack of studies on potential alterations in calcium currents, represents critical shortcomings that warrant further investigation.

The multifaceted effects of SGAs on cardiac ion channels can predispose individuals to QT prolongation via I_Kr_ blockade while concurrently limiting arrhythmia-triggering mechanisms by suppressing certain depolarizing currents. Notably, although clozapine effectively inhibits seven distinct cardiac ion currents, the similarity of these inhibitions results in no significant change in the overall duration of the AP, thereby minimizing QT prolongation [51]. Thus, despite its inhibition of the hERG channel, clozapine has demonstrated a relatively safe cardiac profile in clinical practice. Previous studies have indicated that while clozapine may prolong the QT interval, it does not significantly increase the incidence of TdP. Similarly, other SGAs affecting multiple ion channels exhibit counter-balancing alterations in ionic currents that may result in a proarrhythmic potential lower than anticipated based solely on hERG channel blockade.

In summary, second-generation antipsychotics primarily impact cardiac repolarization through I_Kr_ channel inhibition. Although they may also modulate Ca^2+^, Na^+^, and other K^+^ channels at elevated doses, their effects at therapeutic plasma concentrations are generally mild and often result in clinically insignificant changes. Nonetheless, the simultaneous multichannel effects of these drugs can lead to unexpected and sometimes serious consequences. In conclusion, while the combined effects of SGAs on ion channels reduce the incidence of serious arrhythmias in the broader population, they may elicit critical symptoms in patients with preexisting cardiac conditions or those concurrently taking multiple QT-prolonging medications. Therefore, ECG monitoring and individualized assessment of arrhythmia risk are recommended during SGA treatment.

## Supplemental data

### Graphical abstract

**Graphical abstract.**
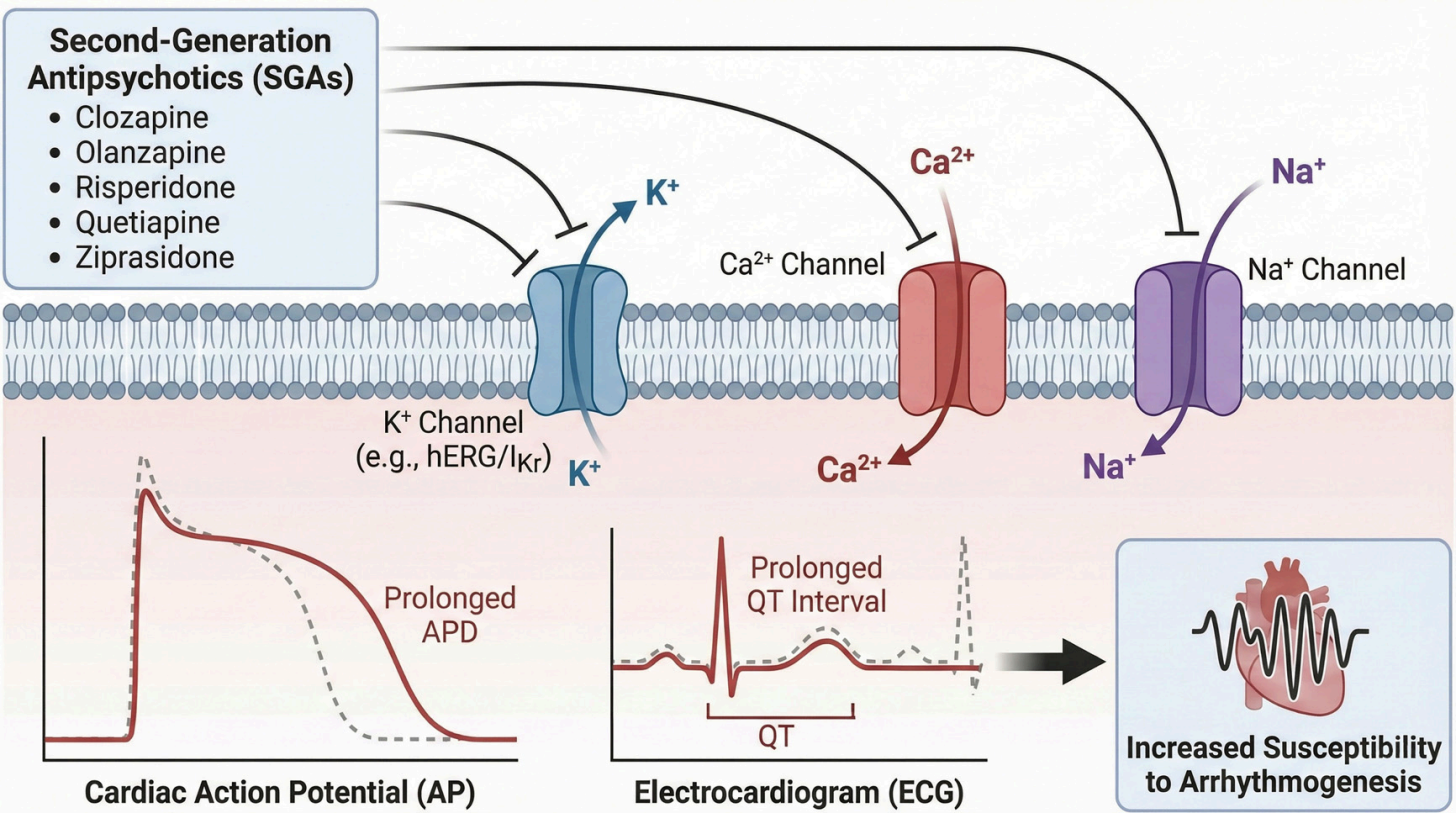
**Schematic representation of the effects of five commonly used second-generation antipsychotic drugs (clozapine, olanzapine, risperidone, quetiapine, and ziprasidone) on cardiac electrophysiology.** These agents influence cardiac K^+^, Ca^2+^, and Na^+^ channels leading to prolongation of action potential duration and the QT interval, thereby increasing susceptibility to arrhythmogenesis.
